# Development and Characterization of a Novel α-Synuclein-PEST H4 Cell Line for Enhanced Drug Screening in α-Synucleinopathies

**DOI:** 10.3390/ijms26157205

**Published:** 2025-07-25

**Authors:** Nancy Carullo, Viktor Haellman, Simon Gutbier, Sonja Schlicht, Thien Thuong Nguyen, Rita Blum Marti, Philippe Hartz, Lothar Lindemann, Lina Schukur

**Affiliations:** 1 Therapeutic Modalities, Pharma Research and Early Development, F. Hoffmann-La Roche Ltd., 4058 Basel, Switzerland; nancy.carullo@roche.com (N.C.);; 2 Neuroscience and Rare Diseases, Pharma Research and Early Development, F. Hoffmann-La Roche Ltd., 4070 Basel, Switzerland

**Keywords:** α-synuclein, Parkinson’s disease, synucleinopathies, drug screening, protein turnover, protein degradation, PEST sequence, cell engineering

## Abstract

Alpha-Synuclein (α-Syn) is a presynaptic neuronal protein implicated in the pathogenesis of Parkinson’s disease (PD) and other synucleinopathies, primarily through its aggregation into insoluble fibrils. The extended α-Syn half-life necessitates treatment durations that are incompatible with efficient high-throughput drug screening, can risk compound stability or cause cellular toxicity. To address this, we inserted a PEST sequence, a motif known to promote rapid protein degradation, at the C-terminus of the *SNCA* gene using CRISPR/Cas9 to create a novel cell line with reduced α-Syn half-life. This modification accelerates α-Syn turnover, providing a robust model for studying α-Syn dynamics and offering a platform that is applicable to other long-lived proteins. Our results demonstrate a six-fold reduction in α-Syn half-life, enabling the rapid detection of changes in protein levels and facilitating the identification of molecules that modulate α-Syn production and degradation pathways. Using inhibitors of the proteasome, transcription, and translation further validated the model’s utility in examining various mechanisms that impact protein levels. This novel cell line represents a significant advancement for studying α-Syn dynamics and offers promising avenues to develop therapeutics for α-synucleinopathies. Future research should focus on validating this model in diverse experimental settings and exploring its potential in high-throughput screening applications.

## 1. Introduction

Alpha-Synuclein (α-Syn) is a small, presynaptic neuronal protein that is highly expressed in the central nervous system, particularly within the neocortex, hippocampus, substantia nigra, thalamus, and cerebellum [1,2]. Although the precise physiological function of α-Syn remains not fully understood, it is thought to be predominantly involved in maintaining synaptic vesicle pools and modulating neurotransmitter release [3,4,5,6].

A major focus of α-Syn research lies in its association with neurodegenerative disorders, in particular Parkinson’s disease (PD) and other synucleinopathies. These disorders present a significant burden on society: in 2021, over 11 million people worldwide had PD and the prevalence is predicted to continue to increase [7,8]. Despite available treatments that alleviate symptoms, there is currently no therapy that can cure, halt, or reverse disease progression. The link between α-Syn and PD was initially established with the identification of mutations in the *SNCA* gene, which encodes the α-Syn protein, in familial forms of PD [9]. Subsequent studies have shown that in PD and other synucleinopathies, α-Syn aggregates into insoluble fibrils, forming Lewy bodies and Lewy neurites, pathological hallmarks of neuronal degeneration [10]. However, the exact mechanisms by which α-Syn contributes to neurodegeneration remain elusive, posing significant challenges for therapeutic intervention.

Investigating α-Syn poses many challenges due to its dynamic nature and propensity to aggregate. Its intrinsically disordered structure allows for diverse interactions with cellular components, complicating efforts to delineate its normal function from its pathological roles. Moreover, the aggregation process is influenced by various factors, including post-translational modifications, genetic mutations, and environmental conditions, making it difficult to predict and control in experimental settings as reviewed in [11]. These complexities have hindered critical drug screening efforts targeting α-Syn in the research and development of therapeutic strategies for PD and α-synucleinopathies. Furthermore, the long half-life of α-Syn introduces multiple obstacles in the drug discovery process, particularly when screening for inhibitors [12,13]. This extended protein presence complicates the assessment of compound efficacy.

In the context of high-throughput screening, long protein half-lives require prolonged incubation and assay durations to effectively observe the impact of potential inhibitors, which can be both time-consuming and costly. Additionally, the stability of the compounds over such extended periods must be ensured to maintain their activity. This necessitates the development of highly sensitive and quantitative assays that can detect subtle changes in protein levels or activity. Moreover, in vivo studies become more challenging as the pharmacokinetics of the inhibitors must be matched to the long half-life of the target protein to achieve sustained inhibition, impacting dosing regimens and interpretation of pharmacodynamic responses. Overall, the long half-life of a target protein requires careful consideration in the design and execution of drug screening strategies.

To address these challenges, we have developed a novel H4 cell line with a reduced α-Syn half-life by inserting a PEST sequence at the 3′ end of the *SNCA* gene using CRISPR/Cas9 technology. The PEST sequence is a distinct motif found within certain proteins that marks them for rapid degradation. It is characterized by a rich presence of proline (P), glutamic acid (E), serine (S), and threonine (T) residues, creating a pattern recognized by cellular degradation systems [14,15]. Proteins containing PEST sequences are typically short-lived and are swiftly targeted by the ubiquitin–proteasome pathway, a primary mechanism for protein catabolism in cells. Here, we capitalize on the properties of the PEST sequence to accelerate the turnover of α-Syn. The reduced half-life enhances assay sensitivity, allowing for a more precise evaluation of compound efficacy and facilitating the identification of drugs that can modulate α-Syn levels. This approach offers new avenues for therapeutic development by rapidly capturing effects on protein levels, thereby addressing challenges such as toxicity associated with prolonged compound incubation times.

In this study, we present the characterization of the α-Syn-PEST cell line and demonstrate its utility in drug screening applications. Our findings revealed that the protein half-life was reduced by about six-fold with the addition of the PEST sequence. We characterized the cell line and discovered that translational and transcriptional modifications can be captured at much earlier time points compared to wild type, with the optimal assay window occurring at 8 h or 16 h. In contrast, changes in wild-type cell lines required longer incubation times, exceeding 24 h, to observe any effects, resulting in a much smaller assay window. Importantly, our data demonstrate that an increased turnover rate of PEST-tagged target proteins can facilitate drug screening efforts. By providing a superior assay window and enhanced sensitivity, this model represents a significant advancement in the quest to understand and target α-Syn in neurodegenerative diseases. This concept serves as a versatile platform, applicable to other proteins with long half-lives, thereby enhancing drug discovery efforts across diverse targets.

## 2. Results

### 2.1. CRISPR/Cas9-Engineered H4 Cell Line Expressing PEST-Tagged α-Syn

**Figure 1:**
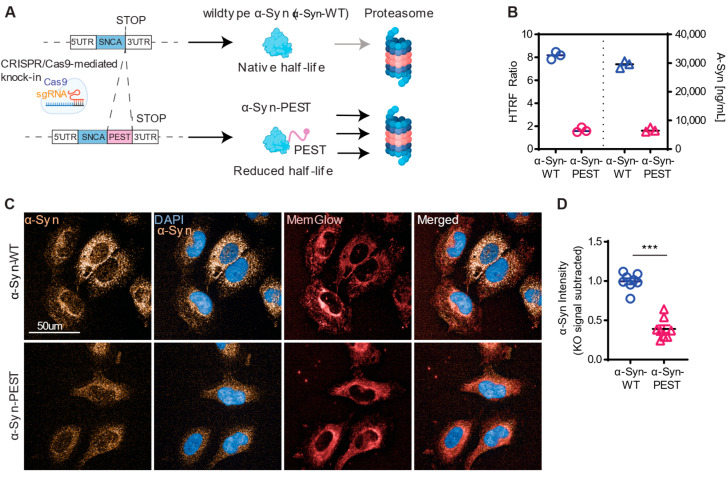
Cell engineering design and verification. (**A**) CRISPR/Cas9-mediated gene editing strategy, illustrating the insertion of the PEST sequence at the 3′ end of the *SNCA* gene in H4 cells to generate an alpha-synuclein (α-Syn)-PEST knock-in cell line. (**B**) Endogenous protein levels in wild-type α-Syn (α-Syn-WT) and α-Syn-PEST cells were determined in a Homogeneous Time-Resolved Fluorescence (HTRF) assay. The HTRF ratio is graphed on the left *y*-axis. The α-Syn concentrations (graphed on the right *y*-axis) were calculated using α-Syn standard. (**C**,**D**) Immunocytochemistry (ICC) of α-Syn-WT and α-Syn-PEST knock-in H4 cells (**C**, example images) confirm the reduced α-Syn protein levels in the α-Syn-PEST line (unpaired *t*-test *t*(14) = 10.02, *** *p* < 0.001) (**D**).

The CRISPR/Cas9 system was employed to insert a PEST sequence at the C-terminus of the *SNCA* gene within H4 neuroglioma cells (Figure 1A). This genetic modification resulted in an endogenous α-Syn protein that is fused to the PEST sequence, which is known to be a signal for rapid degradation via the proteasomal pathway [14,15,16]. Consequently, the half-life of α-Syn in these cells is expected to be significantly reduced, enabling the detection of changes in α-Syn levels within shorter time frames. To ensure consistent degradation dynamics, single-cell clones with a homozygous integration of the PEST sequence were preferred, as a residual wild-type α-Syn would exhibit its native, longer half-life, potentially interfering with assay accuracy and reproducibility. The selected clone, identified based on its optimal signal-to-background ratio using homogeneous time-resolved fluorescent (HTRF) assay, underwent next-generation sequencing (NGS). Our results confirmed the accurate and homozygous insertion of the PEST sequence at the C-terminus of the *SNCA* gene locus (Supplementary Figure S1). Quantitative analysis using HTRF protein assay (Figure 1B) and Immunocytochemistry (ICC, Figure 1C,D) demonstrated a significant reduction in the overall protein levels of the PEST-tagged α-Syn (α-Syn-PEST) compared to its wild-type (WT) counterpart. This reduction is likely attributed to the accelerated degradation of the PEST-tagged form of α-Syn, reflecting a high turnover rate. Importantly, ICC staining comparing α-Syn-WT to α-Syn-PEST cells indicated that the intracellular distribution of α-Syn remained unaffected by the insertion of the PEST sequence (Supplementary Figure S2).

### 2.2. In Vitro Validation of Rapid α-Syn Protein Turnover

**Figure 2:**
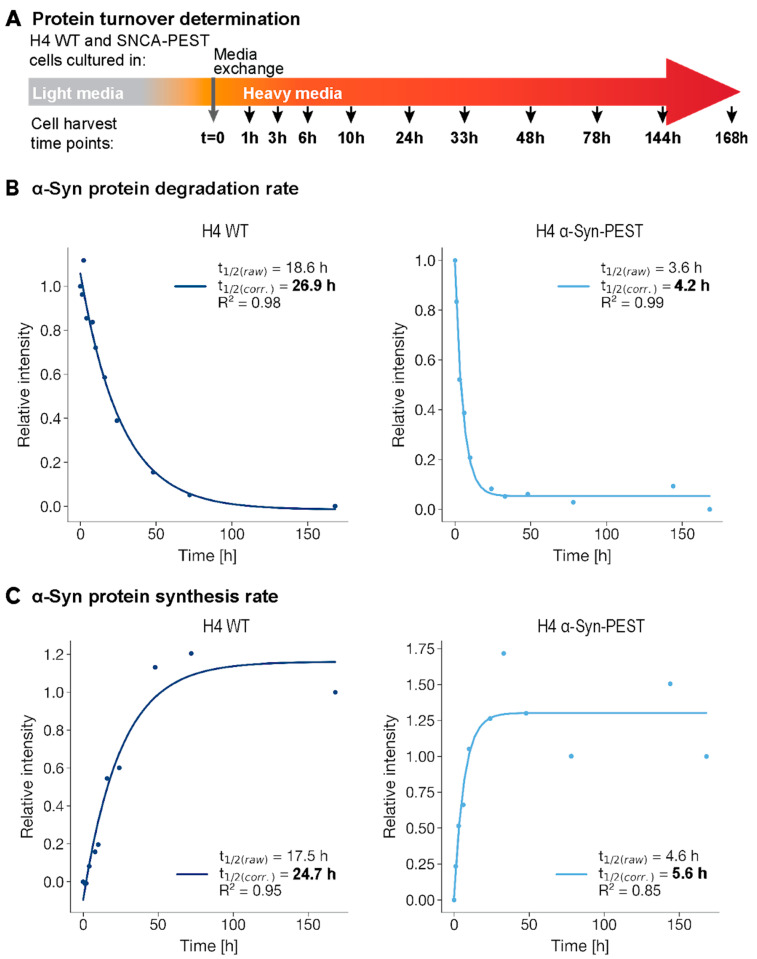
α-Syn half-life characterization using Stable Isotope Labeling by Amino acids in Cell culture (SILAC). (**A**) Experimental design: α-Syn-WT and α-Syn-PEST cells were cultured in light SILAC media (Arg0, Lys0) or heavy SILAC media (Arg10, Lys8). Samples were collected at the indicated time points and processed for LC-MS/MS. (**B**) The light channel indicates the degradation rate, while the heavy channel (**C**) indicates the apparent protein synthesis rate. The measured α-Syn half-life was 26.9 h in WT and 4.2 h in α-Syn-PEST cells. The protein synthesis rate in WT was 24.7 h and 5.6 h in α-Syn-PEST cells.

To investigate the effect of the PEST fusion on the half-life of α-Syn, we employed Stable Isotope Labeling by Amino acids in Cell culture (SILAC) and compared both α-Syn-PEST and α-Syn-WT H4 cells (Figure 2A). This assay utilizes light and heavy isotopic channels to differentiate between protein degradation and synthesis rates [17]. It involves incorporating stable isotope-labeled amino acids into proteins, allowing for the measurement of protein abundance changes under different conditions. In SILAC, one cell population incorporates the ‘heavy’ isotopes of amino acids, while another uses ‘light’ isotopes. Mixed and analyzed by mass spectrometry, the proteins are quantified by the mass difference between isotopes, shedding light on their apparent synthesis and degradation dynamics in response to various stimuli [17]. In our study, cells were cultured in ‘light’ SILAC medium (Arg0, Lys0), transferred to ‘heavy’ SILAC medium (Arg10, Lys8) at the beginning of the experiment (t = 0), and harvested at the indicated time points (Figure 2A). Lysates, prepared with SDS-based lysis buffer, were digested and TMT-labeled before being fractionated into 24 fractions using basic reverse-phase (bRP) chromatography. LC-MS/MS analyses were performed on an Orbitrap QE plus instrument (Thermo Fisher Scientific, Waltham, MA, USA). Data obtained from the light channel provided insights into the degradation rate of α-Syn protein (Figure 2B), demonstrating a markedly accelerated degradation of the PEST-tagged α-Syn compared to its wild-type form. The half-life was reduced by approximately six-fold from 26.9 h in WT to 4.2 h in α-Syn-PEST cells (Figure 2B), consistent with the accelerated apparent synthesis rate (Figure 2C). This finding corroborates the results of reduced α-Syn protein levels presented in Figure 1. In conclusion, the data obtained through the SILAC technique indicate a decrease in the overall level of α-Syn protein and a six-fold decrease in its half-life, confirming the effect of the PEST sequence.

### 2.3. Evaluating the Effect of Translational Inhibition on Short-Lived α-Syn Expression

**Figure 3:**
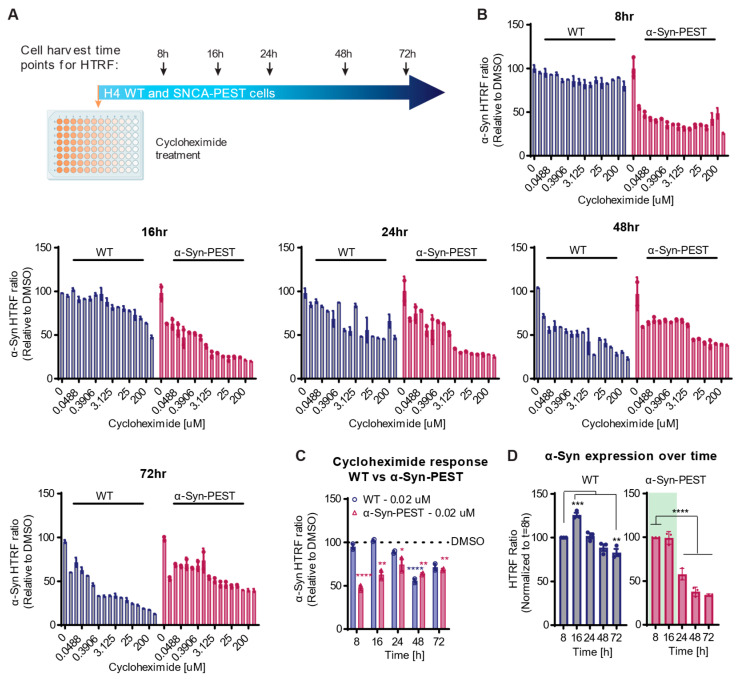
Reduced half-life allows for early detection of protein level modifications. (**A**) Experimental design: α-Syn-WT and α-Syn-PEST H4 cells were treated with increasing doses of the protein translation inhibitor Cycloheximide. Cells were harvested and protein levels were determined in an HTRF assay at the five indicated time points. (**B**) The data was quantified and the resulting HTRF ratios were normalized to the DMSO control, establishing the baseline as 100%. (**C**) The response to 0.02 µM Cycloheximide treatment compared to DMSO over time in both α-Syn-WT and α-Syn-PEST H4 cells. Statistical significance calculated for each time point compared to the respective DMSO control and cell line using two-way ANOVA with Dunnett post hoc test *F*(12, 22) = 4.316, *p* = 0.0015. (**D**) α-Syn protein levels were measured at the indicated time points and normalized to 8 h, respectively (one-way ANOVA with Dunnett post hoc test for WT: *F*(4, 10) = 29.33, *p* < 00001, and α-Syn-PEST *F*(4, 10) = 123.9, *p* < 0.0001). Data expressed as mean ± s.e.m.; *p* values indicated as follows * *p* < 0.05, ** *p* < 0.01, *** *p* < 0.001, **** *p* < 0.0001.

To elucidate the impact of the engineered cell line with a short-lived α-Syn, we used Cycloheximide, a translational inhibitor known to suppress general protein synthesis [18,19]. This inhibitor allowed us to evaluate the decline in existing α-Syn levels without interference from newly synthesized proteins. By comparing the two cell lines, α-Syn-WT and α-Syn-PEST H4 cells, we evaluated the influence of Cycloheximide on α-Syn protein levels in the context of the reduced protein half-life. To do so, we exposed both cell lines to various concentrations of Cycloheximide for 8, 16, 24, and 48 h (Figure 3A). Protein levels were quantified using an HTRF assay, with HTRF ratios of Cycloheximide-treated cells normalized to DMSO-treated cells at each respective time point. In the α-Syn-PEST cell line, reductions in protein levels were apparent after just 8 h of Cycloheximide incubation, whereas α-Syn-WT cells showed no evident change at that time point (Figure 3B). In WT cells, a reduction in α-Syn became notable after 24 h, reaching a maximum decrease of 50%, consistent with the approximately 27 h half-life of α-Syn-WT determined by SILAC (Figure 2). After 48 h and 72 h, we observed a more pronounced decrease in α-Syn-WT levels, while in the α-Syn-PEST cells with the shorter half-life, the effect of Cycloheximide relative to untreated cells began to plateau, suggesting a narrowing assay window (treated/untreated; signal/background, Figure 3C). Importantly, these data indicate that at early time points, which enable a more efficient screening setup (8–16 h), the α-Syn-PEST cell line provides a significantly superior assay window even at low doses (0.02 µM) of Cycloheximide (Figure 3C).

In the next step, we sought to understand the dynamics of α-Syn expression level in both α-Syn-WT and α-Syn-PEST cells over a 72 h period. We analyzed the HTRF ratio in both cell lines at the indicated time points (Figure 3D), normalizing the measured value to the initial time point (t = 8 h). We observed that the total α-Syn level in PEST-tagged cells significantly decreased between the 24–72 h time points (Figure 3D), which may contribute to the narrower assay window. These findings suggest that the optimal period for assessing changes in α-Syn protein levels in the a-Syn-PEST cell line is within the first 16 h. However, we acknowledge that the observed effects are specific to the compound used in this assay.

### 2.4. Detection of Rapid α-Syn Reduction and Rescue

**Figure 4:**
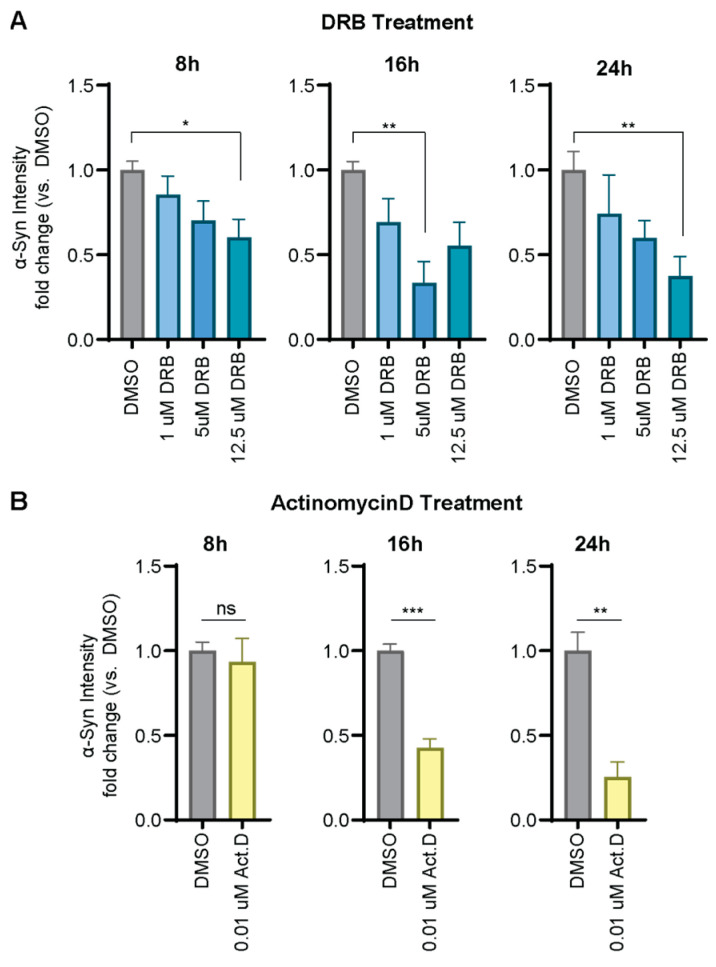
Transcriptional inhibition. Quantification of ICC images of α-Syn protein levels normalized to DMSO-treated α-Syn-PEST cells at each time point. α-Syn protein levels decrease within 8–16 h of treatment with transcriptional inhibitors (**A**) 5,6-Dichloro-1-beta-D-ribofuranosylbenzimidazole (DRB, Welch’s ANOVA with Dunnett post hoc test for 8 h: *n* = 6, *F*(3, 10.3) = 4.386, *p* = 0.0314, for 16 h: *n* = 8, *F*(3, 9.895) = 9.219, *p* = 0.0032, for 24 h: *n* = 6, *F*(3, 10.89) = 4.924, *p* = 0.0211) or (**B**) Actinomycin D (Act.D, Mann–Whitney test for 8 h: *n* = 6, *U* = 17, *p* = 0.9091, for 16 h: *n* = 8, *U* = 0, *p* = 0.0002, for 24 h: *n* = 6, *U* = 0, *p* = 0.0022). Data expressed as mean ± s.e.m. *p* values indicated as follows * *p* < 0.05, ** *p* < 0.01, *** *p* < 0.001.

To explore the broader range of applicability of the α-Syn-PEST cell line, we assessed its suitability to detect transcriptional inhibition. Therefore, we treated the cells with transcriptional inhibitors DRB (5,6-Dichloro-1-beta-D-ribofuranosylbenzimidazole), which targets RNA polymerase II, and Actinomycin D, which intercalates DNA to prevent RNA polymerase movement. α-Syn-PEST cells were exposed to varying concentrations of these compounds (Figure 4A,B) and α-Syn levels were assessed using ICC, normalized to DMSO-treated control cells. Similar to findings with the translational inhibitor Cycloheximide (Figure 3), we observed changes after 8–16 h of treatment and trending dose-dependent reductions in α-Syn expression with increasing concentrations of transcriptional inhibitors (Figure 4A,B). These data demonstrate the utility of the α-Syn-PEST cell line as a valuable tool for studying and screening effects on protein levels through distinct mechanisms.

**Figure 5:**
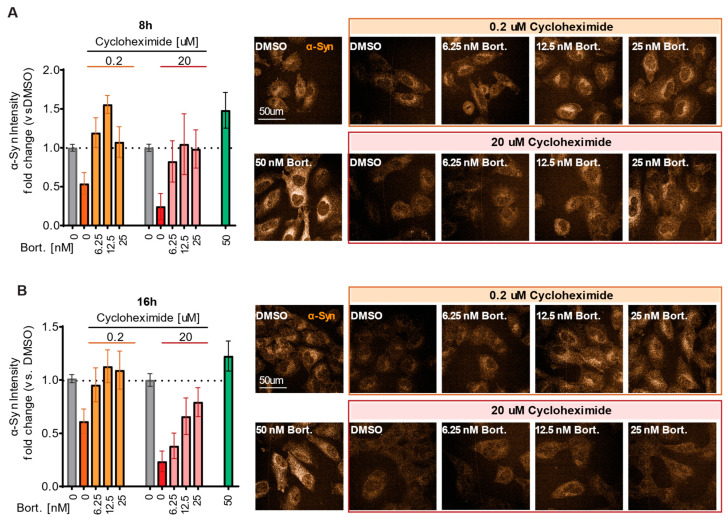
Translational inhibition and rescue of α-Syn in α-Syn-PEST cells. Experimental design: α-Syn-PEST H4 cells were treated with Cycloheximide and increasing doses of the proteasome inhibitor Bortezomib. α-Syn protein levels were quantified and normalized to DMSO controls with ICC images (right) across two time points: (**A**) 8 h (Welch’s ANOVA *n* = 8, *F*(9, 27.74) = 5.219 *p* = 0.0004), (**B**) 16 h (Welch’s ANOVA *n* = 8, *F*(3, 27.88) = 8.287, *p* < 0.0001). Data expressed as mean ± s.e.m.

To determine whether the differentiated α-Syn reduction in the α-Syn-PEST cell line compared to its wild-type counterpart is driven by proteasomal degradation, we exposed the cells to a dual-treatment approach using Cycloheximide and Bortezomib, a known proteasome inhibitor. Cells were treated with increasing concentrations of Bortezomib (6.25–50 nM) alongside high (20 µM) or low (0.2 µM) concentrations of Cycloheximide (Figure 5). DMSO and 50 nM Bortezomib treatments were used as negative and positive controls, respectively. The aim of this experiment was to determine whether Bortezomib could counteract the Cycloheximide-induced reduction in α-Syn. Notably, Bortezomib treatment led to a rescue of α-Syn levels as early as 8 h (Figure 5A) and 16 h (Figure 5B). As expected, our data indicate that a rapid reduction in α-Syn was driven by the proteasomal degradation pathway.

In conclusion, our experiments demonstrate that the engineered α-Syn-PEST cell line effectively detects translational and transcriptional changes at early time points, and that these alterations can be reversed through proteasomal inhibition.

## 3. Discussion

Long protein lifespans increase the susceptibility to aggregation and misfolding, as supported by the existing literature [20,21,22]. This phenomenon poses significant challenges to the study of long-lived proteins and substantially complicates drug discovery efforts. α-Syn is a prime example; its prolonged half-life and propensity to form toxic oligomers and fibrils render it particularly difficult to investigate and therapeutically address [11,12,23]. Detecting changes in proteins with slow turnover rates, such as α-Syn, often necessitates extended exposure times to experimental compounds, which frequently proves impractical in high-throughput drug screens.

To address these limitations, various cell-based models, including α-Syn fusion proteins with reporters like luciferase or GFP, have been developed [12,23]. However, these systems often rely on the ubiquitous overexpression of α-Syn, which lacks native regulatory elements like the promoter and other regulatory sequences, potentially limiting assay utility. Furthermore, the addition of large tags in fusion proteins has the potential to perturb the physiological characteristics of target proteins, leading to false results [24,25]. Finally, there remains a risk of identifying artifacts arising from antagonistic or agonistic effects on reporters with enzymatic activity, or from interferences with fluorescent signals [26,27,28,29]. Thus, alternative models using short-lived α-Syn variants, which can capitalize on the rapid turnover to facilitate the detection of compounds that modulate protein expression, are highly desirable.

Building upon this rationale, we developed a novel α-Syn-PEST H4 cell line using CRISPR/Cas9 technology to insert a PEST sequence, resulting in a six-fold reduction in α-Syn’s half-life. This accelerated protein turnover transforms the cell line into a highly sensitive tool for identifying compounds that modulate α-Syn production or clearance. Changes in protein levels can be detected in significantly shorter timeframes, as evidenced by Cycloheximide experiments where reductions were observed within just 8 h, compared to 24 h in the WT cells. Notably, the α-Syn-PEST H4 cell line was generated from a sequence-verified single clone. This step is crucial to avoid potential biases from CRISPR/Cas9 off-target effects or the variability of protein degradation rates within a mixed cell population. However, it is important to acknowledge that findings from this rapid turnover system should ideally be further validated in a WT α-Syn cell model to assess their relevance to physiological conditions and improve in vivo translatability.

The utility of the α-Syn-PEST cell line extends beyond conventional drug screening assays. Our experiments involving transcription, translation, and proteasome inhibitors further substantiate its broad applicability in studying diverse mechanisms affecting protein abundance. Ultimately, this would allow for the identification of compounds that affect processes upstream of protein translation or degradation, which may be promising targets given the importance of α-Syn homeostasis [30]. The dose-dependent recovery of α-Syn levels following compound treatment further highlights the potential of this model to investigate the nuanced effects of various compounds on α-Syn dynamics.

It is worth comparing our approach with a recently developed model by Casalino et al., which employs an SH-SY5Y cell line expressing a short-lived α-Syn-luciferase-PEST fusion protein [12]. While this fusion protein model offers the benefit of combining rapid protein turnover with a readily screenable luciferase reporter, it inherently carries the limitation that the larger fusion protein may not accurately recapitulate the behavior of endogenous α-Syn, potentially leading to false positive or false negative data. In contrast, our cell line directly measures changes in endogenous α-Syn, providing insights more aligned with its actual dynamics. Specifically, the α-Syn sequence in our model is maintained as close as possible to the native protein, with only a PEST sequence inserted at the C-terminus to confer rapid degradation. This avoidance of a bulky reporter tag significantly reduces the risk of artifacts such as altered aggregation or mislocalization, which can arise from tag-induced perturbations. While our initial observations did not reveal substantial changes in protein localization and distribution within cells (Supplementary Figure S2), it remains important to acknowledge the possibility that the PEST sequence insertion may exert other, presently uncharacterized effects on the protein. To effectively use the engineered cell line for addressing biological and pathobiological questions related to α-Syn, further investigation is needed to verify that the short-lived α-Syn-PEST can recapitulate the protein’s native role. Beyond the short half-life, it is possible that the lower baseline levels of α-Syn or the increased load on the proteasome influence the cell line’s physiology or its response to certain stimuli.

In conclusion, despite the limitations inherent to every experimental model, the α-Syn-PEST H4 cell line represents a powerful tool for advancing our understanding of α-Syn dynamics, especially in response to potential therapeutic compounds for neurodegenerative diseases. By providing a more physiologically relevant model with enhanced sensitivity and a more pronounced assay window at early time points, this cell line holds substantial promise for accelerating the discovery and development of therapeutic strategies targeting α-synucleinopathies. Future studies should undoubtedly focus on systematically exploring its potential in high-throughput screening applications, and on further characterizing the detailed mechanisms of α-Syn turnover and homeostasis within this engineered cell line.

## 4. Materials and Methods

### 4.1. Cell Culture and Transfection

The following cell line (H4, Cat# HTB-148, ATCC, Manassas, VA, USA) was maintained under standard conditions (37 °C, 5% CO_2_) and cultured in DMEM (high glucose, GlutaMAX, and pyruvate, Cat# 31966, Thermo Fischer Scientific, Waltham, MA, USA) supplemented with 10% Fetal Bovine Serum (Gibco, OneShot A5670402, Thermo Fisher Scientific, Waltham, MA, USA) and 1% Penicillin/Streptomycin. The single guide RNA (sgRNA) used for the CRISPR/Cas9-mediated knock-in (AAAGATATTTCTTAGGCTTC) was designed and ordered from Synthego, Redwood City, CA, USA, and was used in combination with the homology template as single-stranded DNA (ssDNA) (5′ > 3′: AGCAGATCTCAAGAAACTGGGAGCAAAGATATTTCTTAGACGTTGATGCGAGCTGAAGCACAAGCGGCTGGGTGCCGGTCCATACCGCTTTCTTGTGCGCAGGACATAGGCAATGTACCGGCGGCTTGCTCTTCAACCTCAGGCGGAAAGCCGTGAGAGGCTTCAGGTTCGTAGTCTTGATACCCTTCCTAATATTAGAA), synthesized by Integrated DNA Technologies (IDT, Coralville, IA, USA), and Cas9 protein (Cat# A36498, Invitrogen, Thermo Fisher Scientific, Waltham, MA, USA). Transfection was carried out by complexing sgRNA, homology template, and Cas9 protein prior to transfection, which was performed using the 4D Nucleofector device (program CM-167). The amount of sgRNA:Cas9:ssDNA was used as a ratio of 2:2:1. The transfected cells represented a pool of cells that were used to select individual clones for sequence verification via NGS, further characterization, and analysis.

### 4.2. HTRF Protein Detection Assay

H4 cells with α-Syn or α-Syn-PEST tag were cultured as described above in 384 well plates (Cat# 3570, Corning, Glendale, AZ, USA) in 20 µL of medium and at a cell density of 20,000 cells per well. After treatment, culture media were removed, and cells were washed twice with PBS. The HTRF assay (Cat# 6FNSYPEG, Revvity, Graefelfing, Germany) was performed according to the manufacturer’s instructions. Fluorescence was measured using PheraSTAR at the following settings: emission: 620 nm, 665 nm; integration start: 10 µs; integration time: 200 µs. The HTRF ratio was calculated using the following equation: (Signal 665/Signal 620) × 10,000. Data were normalized to control cells as described in the figure legends or results section at the indicated time points.

### 4.3. Protein Half-Life Determination Using SILAC

H4 cells with α-Syn or α-Syn-PEST tag were used to assess the half-life of α-Syn, respectively. Therefore, cells were cultured in ‘light’ SILAC medium (Arg0, Lys0) and transferred to ‘heavy’ SILAC medium (Arg10, Lys8) at the beginning of the experiment (t = 0). Lysates (SDS-based lysis buffer) were then collected at different time points, digested, and TMT-labeled before fractionation via basic reverse-phase (bRP) chromatography into 24 fractions.

Cell pellets were lysed using a lysis buffer consisting of 5% SDS, 50 mM TEAB (pH 7.5). Total protein was determined using a BCA assay (Thermo Fisher Scientific, Waltham, MA, USA) according to the manufacturer’s instructions. Proteins were further digested into peptides using the suspension trapping approach [31] with 1:25 trypsin–protein at 37 °C overnight. Peptides were desalted by C18 solid-phase extraction (using the Bravo AssayMap platform with C18 cartridges) and dried in vacuo. TMT11 labeling was performed according to [32]; equal amounts were combined per TMT plex and desalted by C18 solid-phase extraction cartridges (50 mg cartridges, Sep-Pak, Waters, Milford, MA, USA). Desalted peptides were chromatographically separated via bRP chromatography into 24 fractions, dried down, and stored at −20 °C until further analysis [33].

The samples were measured by nLC-MS/MS system (NanoLC: UltiMate3000; MS: Orbitrap QE plus; both Thermo Fisher Scientific, Waltham, MA, USA) with the following settings: 120 min nLC gradient, data-dependent acquisition, fragmentation method: higher energy collisional dissociation (HCD). Peptides and proteins were identified and quantified using MaxQuant (Version: 1.6.2.6), with the following settings: protease: Trypsin; fixed modifications: Carbamidomethyl (C); variable modifications: Oxidation (M); Acetyl (Protein N-term); Arg10 (R); Lys8 (K); protein sequence database: Human SwissProt Database; PSM/Protein FDR: 1%

### 4.4. Immunocytochemistry

H4 cells with α-Syn or α-Syn-PEST tag were cultured as described above in 384 well plates (Cat# 3570, Corning). Following treatment with Cycloheximide (Cat# C7698, Sigma-Aldrich, St. Louis, MO, USA) and Bortezomib (Cat# ab142123, Abcam, Cambridge, MA, USA), DRB (Cat# FBM-10-2047, Lucerna-Chem AG, Luzern, Switzerland), and Actinomycin D (Cat# ab291108, Abcam, Cambridge, MA, USA) with the concentrations and treatment durations indicated in Figure 3, Figure 4 and Figure 5, paraformaldehyde (PFA, Cat# 15710, Electron Microscopy Sciences, Hatfield, PA, USA) was added to the media to reach a final concentration of 4% PFA. Cells were fixed for 15 min, then washed three times in PBS. Following 15 min of permeabilization (PBS with 0.3% Tween), the cells were rinsed with PBS and blocked in 1x Animal-free blocking solution (Cat# SP-5030, Vector laboratories, Newark, CA, USA) in PBS with 0.1% Tween for 30 min. The cells were incubated with the primary antibody (α-Synuclein Mouse, Cat# 610787, BD, Franklin Lakes, NJ, USA) at a 1:200 dilution at 4 °C overnight. Following three PBS wash steps, cells were incubated with a secondary antibody solution containing 1:1000 secondary antibody (goat anti-mouse AF555, Cat# A32727, Invitrogen, Thermo Fisher Scientific, Waltham, MA, USA) with DAPI (1:1000 dilution) for 1 h at room temperature. Following two PBS wash steps, cells were incubated with a 1:1000 dilution of MemGlow™ 640 (Cat# MG04, Cytoskeleton, Denver, CO, USA) for 15 min and washed twice in PBS prior to imaging. The cells were then imaged in PBS on a Perkin-Elmer Operetta CLS microscope system and analyzed in Harmony^®^ 4.9. For the image analysis, DAPI and Memglow stainings were used to identify nuclei and cell outlines. α-Syn signal intensity was quantified within cell outlines. The data was normalized to the respective DMSO control groups for each cell line and time point as indicated.

### 4.5. Statistical Analysis

Statistical analyses and graphs were generated using GraphPad Prism version 10. Appropriate statistical tests were chosen and the required assumptions were evaluated and tested in reference to [34]. Briefly, to statistically compare two groups, the normal distribution of the data was first assessed. An F-test was employed to compare variances between two groups. If all assumptions were met, an independent Student’s t-test was applied; otherwise, the nonparametric Mann–Whitney U-test was applied instead. To compare the means between three groups, the normal distribution of the data between groups was assessed and standard variance was determined using the Browne–Forsythe test. When all assumptions were met, an ordinary one-way ANOVA test was applied; otherwise, the Welch’s ANOVA test was used instead. If the ANOVA test revealed a statistical difference between means, Dunnett’s multiple comparison test was used to determine which specific groups were statistically different. To compare how a response is affected by two factors, the normal distribution of the data was assessed. A Shapiro–Wilk test was used to assess the normality of the residuals. When all assumptions were met, an ordinary two-way ANOVA was performed. If the two-way ANOVA revealed a statistical difference between means, Sidak’s multiple comparison test was used to determine which specific groups were statistically different. Detailed information about the specific tests used for each data set can be found in the respective figure captions.

## Data Availability

The raw data supporting the conclusions of this article will be made available by the authors upon request.

## Supplementary Materials

The supporting information can be downloaded at https://www.mdpi.com/article/10.3390/ijms26157205/s1.

## Abbreviations

The following abbreviations are used in this manuscript:

α-Syn	Alpha-Synuclein
PEST	Proline, glutamic acid, serine, threonine
PD	Parkinson’s disease
NGS	Next-generation sequencing
ICC	Immunocytochemistry
α-Syn-PEST	PEST-tagged α-Syn
WT	Wild type
SILAC	Stable Isotope Labeling by Amino acids in Cell culture
bRP	basic reverse-phase
HTRF	Homogeneous Time-Resolved Fluorescence
Bort.	Bortezomib
DRB	5,6-Dichloro-1-beta-D-ribofuranosylbenzimidazole
Act.D	Actinomycin D
