# Supplementary material for: Development and Characterization of a Novel α-Synuclein-PEST H4 Cell Line for Enhanced Drug Screening in α-Synucleinopathies
Source: Int J Mol Sci. 2025 Jul 25;26(15):7205. doi: 10.3390/ijms26157205 (PMC12346604; doi:10.3390/ijms26157205)
Supplement: Supplementary file 1 [file ijms-26-07205-s001.zip › ijms-3741536-supplementary.pdf]

PEST sequence

Nucleotide sequence: TCTCACGGCTTTCCGCCTGAGGTTGAAGAGCAAGCCGCCGGTACATTGCCTATGTCCTGCG
CACAAGAAAGCGGTATGGACCGGCACCCAGCCGCTTGTGCTTCAGCTCGCATCAACGTC

Amino acid sequence: SHGFPPEVEEQAAGTLPMSCAQESGMDRHPAACASARINV

NGS Sequencing Results

```
                 Consensus ACTAGGTATATGATCATTAAATGGTGCATCCGGATCAGAACCTAGATATTTTAACTCTGACTACTACTGTAATTCACTTT  80
α-Syn-PEST single cell clone ................................................................................  80
                  α-Syn-WT ................................................................................  80

                 Consensus TATATCAGACAAGAAAGACACAACTATTAAAAATAAGATAATATTTGCTGCAGAATATTTGCAAAAACATTGATTGTAAA 160
α-Syn-PEST single cell clone ................................................................................ 160
                  α-Syn-WT ................................................................................ 160

                 Consensus TTTTAGTGTAAGTGGGGAGCCATTTCCTATCTCATTGGCTGTCAGTGCTGATGCGTAATTGAAACTTATACTAACAGTGT 240
α-Syn-PEST single cell clone ................................................................................ 240
                  α-Syn-WT ................................................................................ 240

                 Consensus GTGCTGTCTTTTTGATTTTTCTAATATTAGGAAGGGTATCAAGACTACGAACCTGAAGCCNNNNNNNNNNNNNNNNNNNN 320
α-Syn-PEST single cell clone ............................................................TCTCACGGCTTTCCGCCTGA 320
                  α-Syn-WT ............................................................-------------------- 300

                 Consensus NNNNNNNNNNNNNNNNNNNNNNNNNNNNNNNNNNNNNNNNNNNNNNNNNNNNNNNNNNNNNNNNNNNNNNNNNNNNNNNN 400
α-Syn-PEST single cell clone GGTTGAAGAGCAAGCCGCCGGTACATTGCCTATGTCCTGCGCACAAGAAAGCGGTATGGACCGGCACCCAGCCGCTTGTG 400
                  α-Syn-WT -------------------------------------------------------------------------------- 300

                 Consensus NNNNNNNNNNNNNNNNNNNNTAAGAAATATCTTTGCTCCCAGTTTCTTGAGATCTGCTGACAGATGTTCCATCCTGTACA 480
α-Syn-PEST single cell clone CTTCAGCTCGCATCAACGTC............................................................ 480
                  α-Syn-WT --------------------............................................................ 360

                 Consensus AGTGCTCAGTTCCAATGTGCCCAGTCATGACATTTCTCAAAGTTTTTACAGTGTATCTCGAAGTCTTCCATCAGCAGTGA 560
α-Syn-PEST single cell clone ................................................................................ 560
                  α-Syn-WT ................................................................................ 440

                 Consensus TTGAAGTATCTGTACCTGCCCCCACTCAGCATTTCGGTGCTTCCCTTTCACTGAAGTGAATACATGGTAGCAGGGTCTTT 640
α-Syn-PEST single cell clone ................................................................................ 640
                  α-Syn-WT ................................................................................ 520

                 Consensus GTGTGCTGTGGATTTTGTGGCTTCAATCTACGATGTTAAAACAAATTAAAAACACCTAAGTGACTACCACTTATTTCTAA 720
α-Syn-PEST single cell clone ................................................................................ 720
                  α-Syn-WT ................................................................................ 600
```

Supplementary Figure S1. Sequence Verification of the α-Syn-PEST cell line
Nucleotide and amino acid sequence of the PEST sequence (top) that was inserted at the C′ end of the *SNCA* gene in H4 cells as verified by next-generation sequencing (NGS, bottom). PEST sequence insertion site: Red lettering indicates the introduced PEST nucleotide sequence. Dots represent alignment, while dashes indicate mismatch with the reference (WT) sequence.

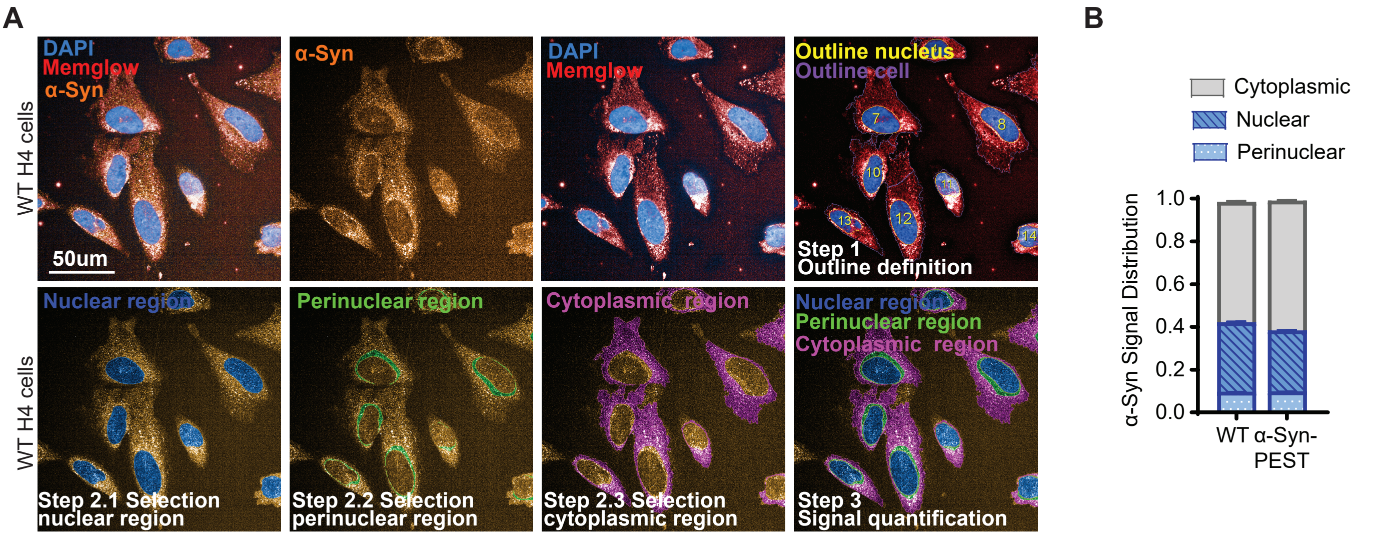

Supplementary Figure S2. ***α*-Syn distribution within cells.** (**A**) For the image analysis, DAPI and Memglow stainings were used to identify nuclei and cell outlines (top panel). *α*-Syn signal intensity was measured in whole cells as well as in three biologically relevant cellular compartments (nuclear, perinuclear, cytoplasmic regions; bottom panel). (**B**) Quantified data showing that the insertion of a PEST sequence into the *SNCA* gene does not alter the protein distribution of *α*-Syn within cells (Two-way ANOVA $F(1, 471) = 0.7577$, $p = 0.3845$). Data expressed as mean ± s.e.m.
